# Sensorineural Hearing Loss in Major Systemic Autoimmune Rheumatic Diseases: A Systematic Review and Meta-Analysis

**DOI:** 10.3390/jcm15166456

**Published:** 2026-08-20

**Authors:** Amer Saffouri, Alaa Safia, Sameer Sawaed, Azzam Azzam, Sohaib Omari, Yassin Rabah, Uday Abd Elhadi

**Affiliations:** 1 Department of Internal Medicine, Hospital Nazareth EMMS, Nazareth 1622222, Israel; 2 The Azrieli Faculty of Medicine, Bar-Ilan University, Safed 1311502, Israel; 3 Head & Neck Surgery Unit, Department of Otolaryngology, Ziv Medical Center, Safed 1311502, Israel; 4 Department of Intensive Care Unit, Ziv Medical Center, Safed 1311502, Israel

**Keywords:** sensorineural hearing loss, systemic autoimmune rheumatic disease, rheumatoid arthritis, systemic lupus erythematosus, primary sjögren syndrome, systemic sclerosis

## Abstract

**Background:** Sensorineural hearing loss (SNHL) has increasingly been recognized as an extra-articular manifestation of systemic autoimmune rheumatic disease (SARDs), but its burden and clinical characteristics remain inconsistent. This systematic review and meta-analysis evaluated the prevalence, risk, audiometric characteristics, diagnostic methods and prognostic factors of SNHL in patients with major SARDs. **Methods:** A systematic search of the PubMed, Scopus and Cochrane Library databases was conducted between 25 June and 3 July 2026 in accordance with PRISMA 2020 guidelines. Observational studies evaluating SNHL in patients with rheumatoid arthritis (RA), systemic lupus erythematosus (SLE), primary Sjögren syndrome (pSS) and systemic sclerosis (SSc) were included. Meta-analyses using a random-effects model were performed to estimate pooled prevalence and odds ratios (OR). Subgroup analyses, meta-regression, sensitivity analysis, publication bias assessment and certainty of evidence evaluation were performed. **Results:** Twenty-two studies met the eligibility criteria and were included. The pooled prevalence of SNHL was 50% (95% CI: 13–87%, *p* < 0.001) in patients with RA, 61% (95% CI: 22–95%, *p* < 0.001) in SLE, 31% (95% CI: 5–67%, *p* < 0.001) in pSS and 28% (95% CI: 14–44%, *p* < 0.001) in SS. Patients with RA (OR 1.92; 95% CI: 1.29–2.87) and SLE (OR 21.68; 95% CI: 4.65–100.99) had significantly increased odds of SNHL compared to healthy controls. Hearing loss was predominantly mild, bilateral, and cochlear in origin, and affected high or extended high frequencies. Longer disease duration and greater disease activity were associated with worse hearing thresholds. In exploratory analyses of RA studies, sample size, study setting, and study design were significant study-level moderators of heterogeneity. **Conclusions:** SNHL is reported with appreciable prevalence across several major systemic autoimmune rheumatic diseases, although prevalence estimates are highly heterogeneous and should be interpreted cautiously. Comparative evidence supports increased odds of SNHL in RA, whereas the magnitude of the association in SLE remains uncertain because of the limited and imprecise evidence. Evidence establishing increased comparative risk in pSS and SSc remains insufficient. Audiological assessment may be particularly relevant in patients with longstanding or active disease.

## 1. Introduction

Sensorineural hearing loss is caused by damage to the cochlea, auditory nerve, or central auditory pathways, resulting in permanent hearing impairment [1]. The condition is categorized as sudden, congenital and progressive hearing loss [1,2,3,4]. The etiology of SNHL is a complex interplay between genetic and environmental factors, with core pathologies like mitochondrial dysfunction and oxidative stress dictating the clinical severity of the condition [5]. The onset of sensorineural hearing loss can vary in children and adults. Sudden SNHL is characterized by an abrupt hearing loss of 30 decibels or greater across at least three consecutive audiometric frequencies over 72 h [6]. In contrast, congenital hearing loss can be present at birth but progressive cases worsen over months or years [7].

The incidence of SNHL varies across countries, with prevalence being high in low- and middle-income countries, particularly in Sub-Saharan Africa and South Asia [2]. Research shows that prevalence of sudden and congenital SNHL is approximately 5–27 per 100,000 people annually and 1–3 per 1000 live births [3,4]. Incidences as high as 160 to 400 per 100,000 people have been reported in Germany [8,9]. Since some sudden SNHL cases recover spontaneously, actual incidence may be higher [3]. Age-related SNHL increases exponentially in up to 25% of adults between the age of 50 and 60 years while noise-influenced SNHL affects approximately 16% of global adults with disabling hearing loss cases [10].

Many studies confirm a strong link between systemic autoimmune diseases and sensorineural hearing loss. Clinical trials have reported an association between SNHL, particularly sudden cases, and major systemic autoimmune rheumatic diseases (SARD) such as rheumatoid arthritis (RA), Cogan’s syndrome, systemic lupus erythematosus (SLE) and vitiligo [11,12]. The pathophysiology behind SNHL in SARDs remains complex, involving a combination of localized inner ear targeting and systemic vascular damage [13]. Due to shared structural epitopes, antibodies in autoimmune diseases can cause cross-reactive molecular mimicry targeting native inner-ear proteins alongside localized micro-vascular thrombosis [14]. Immune complexes in RA, SLE or antiphospholipid syndrome (APS) form in the endothelium of microvascular network inducing local vasculitis and micro-infarctions causing the destruction of hair cells within the inner ear [15]. SNHL is a frequently encountered audiovestibular symptom in SARDs although the prevalence is inconsistent reported at between 6% and 70% [16]. Studies have reported that approximately 25–72% of RA patients and 7–8% of vitiligo patients experience SNHL [17,18].

While several systematic reviews have summarized the incidence of SNHL in SARDs, quantitative evidence remains scarce and largely restricted to selected diseases, particularly RA and SLE [19]. The relative risk of SNHL in major SARDs has not been comprehensively investigated. Further, evaluation of diagnostic methods, audiometric characteristics and prognostic factors that may influence hearing outcomes in patients with SARDs is underdeveloped. This systematic review and meta-analysis evaluated the prevalence of SNHL in rheumatoid arthritis (RA), systemic lupus erythematosus (SLE), primary Sjogren syndrome (pSS) and systemic sclerosis (SSc). The review evaluated the odds of SNHL in patients compared to healthy controls, and examined audiometric, diagnostic and prognostic factors associated with SNHL.

## 2. Methods

This systematic review and meta-analysis was conducted in conformance with the Preferred Reporting items of Systematic Reviews and Meta-Analysis (PRISMA; Table S1) 2020 guidelines [19]. The study protocol was registered on PROSPERO (CRD420261457883).

### 2.1. Search Strategy

A comprehensive literature search was conducted in PubMed, Scopus and Cochrane Library databases between 25 June and 3 July 2026. Keywords and MeSH terms related to sensorineural hearing loss, systemic autoimmune rheumatic disease, rheumatoid arthritis, systemic lupus erythematosus, primary Sjogren syndrome and systemic sclerosis were used in the databases to locate relevant articles. The MeSH terms were: (“Sensorineural Hearing Loss” [MeSH] OR “Hearing Disorder” [MeSH] OR “Audiometry” [MeSH]) AND (“Systemic Autoimmune Rheumatic Disease” [MeSH] OR “Rheumatoid Arthritis” [MeSH] OR “Systemic Lupus Erythematosus” [MeSH] OR “Primary Sjogren Syndrome” [MeSH] OR “Systemic Sclerosis” [MeSH]). The search query was tailored to each database syntax. The search was limited to English studies but there was no restriction on year of publication.

### 2.2. Inclusion and Exclusion Criteria

Randomized controlled trials, prospective and retrospective cohort studies were included in the review. The studies reported prevalence of sensorineural hearing loss in RA, SLE, pSS and SSc. In addition, the studies reported odds of SNHL in patients compared to healthy controls, and examined audiometric, diagnostic and prognostic factors associated with SNHL. Only English studies were considered for review. Studies that did not report prevalence or odds ratios (OR) of SNHL were excluded. Case studies, in vitro studies, conference abstracts, case reports and non-English studies were also excluded from the review.

### 2.3. Screening of Studies and Data Extraction

Two authors independently screened the titles and abstracts of the identified articles for relevance. A screening of the full texts of the retrieved studies against the inclusion and exclusion criteria was conducted to identify eligible studies for inclusion in the review. Disagreements were resolved through consultation or consensus.

A standardized excel template was used to extract data from the selected studies. The data extracted included author, year, study design, sample size, mean age, gender, SNHL type, SARD type, and treatment duration. Outcome data included reported effect estimates (odds ratio, risk ratio, hazard ratio, or relative risk), audiometric findings (speech audiometry, pure-tone audiometry thresholds, hearing thresholds), diagnostic assessments (vestibular function tests, tympanometry, and auditory brain response), characteristics of hearing loss (severity, laterality, frequency distribution) and prognostic factors (disease duration, disease activity indices, and treatment exposure).

### 2.4. Data Analysis

A meta-analysis was conducted using R software, version 4.5.1 (R Foundation for Statistical Computing, Vienna, Austria). Pooled prevalence of SNHL was calculated separately for RA, SLE, pSS and SSc. Similarly, pooled odds ratios (OR) for SNHL in patients versus healthy controls were also calculated. The pooled analyses were calculated using random-effects model with 95% confidence intervals and heterogeneity assessed using *I*^2^-statistic and Cochran’s Q test. A sensitivity analysis was performed using a leave-one-out approach to assess the influence of individual studies on pooled prevalence and odds ratio estimates. Publication bias was assessed using funnel plots and Egger’s test. The Grading of Recommendations Assessment, Development and Evaluation (GRADE) framework was used to assess the certainty of evidence for outcomes across domains, including risk of bias, inconsistency, imprecision and publication bias. Certainty was defined as very low, low, moderate and high.

### 2.5. Quality Assessment

The Newcastle–Ottawa Scale (NOS) was used to assess risk of bias of observational studies across comparability, patient selection and outcome assessment domains. Overall methodological quality was defined as high (7–9 stars), moderate (4–6 stars) or low (0–3 stars) quality [20].

## 3. Results

**Figure 1:**
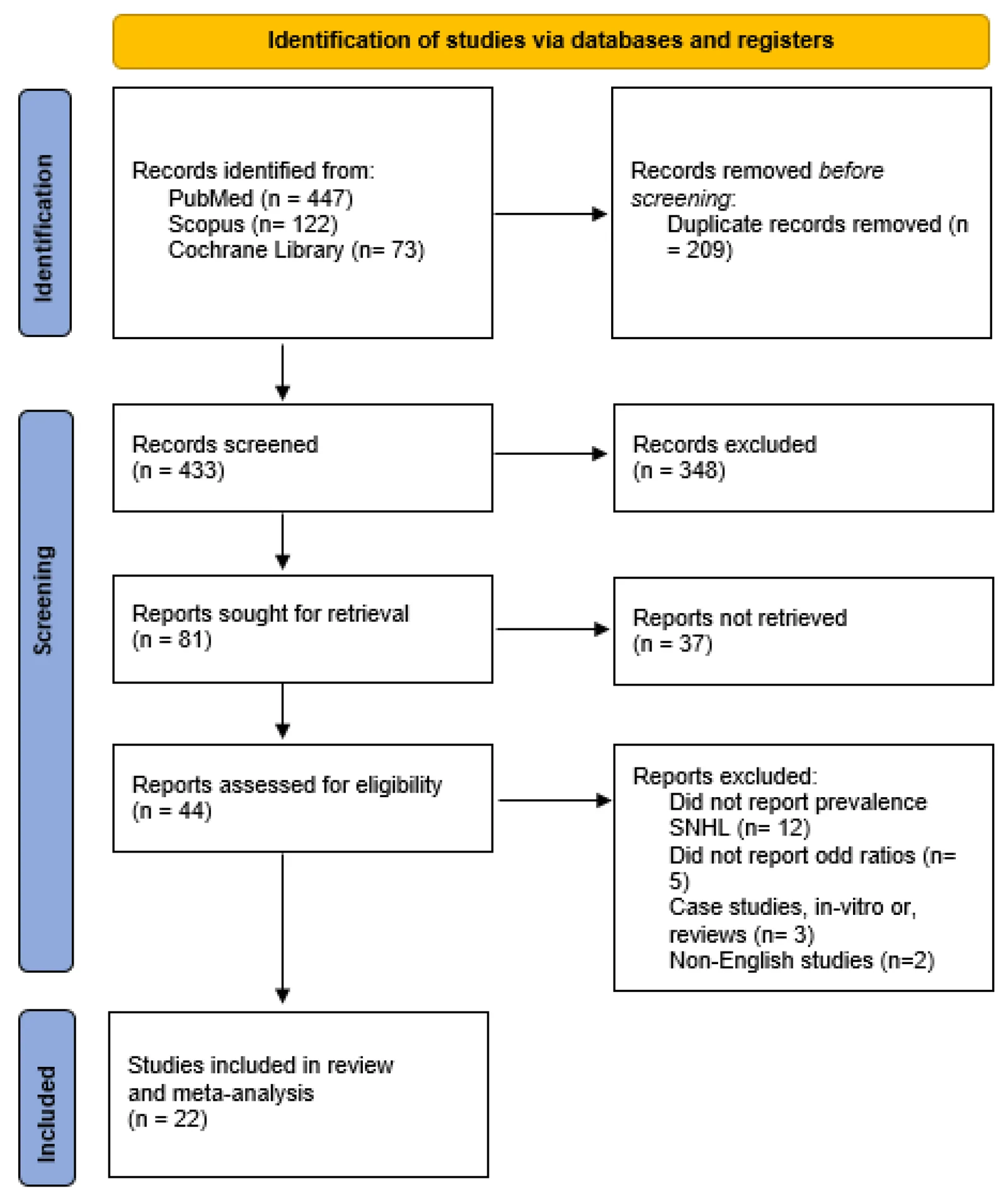
PRISMA flow chart illustrating selection of included studies.

The initial search in the databases generated 642 studies, of which 209 duplicates were excluded. A screening of the titles and abstracts of the remaining 433 articles led to the exclusion of 348 records. Out of the 81 articles sought for retrieval, 37 articles were closed-access and hence excluded. The full texts of the remaining 44 articles were screened and assessed for eligibility; 12 articles that did not report prevalence of SNHL, five that did not report odd ratios of SNHL, three case studies, in vitro studies or review studies, and two non-English studies were excluded. Only 22 articles fully met the inclusion criteria and were included in the review. Figure 1 shows the PRISMA flow chart illustrating the selection of the included studies.

### 3.1. Study Characteristics

A total of 22 cross-sectional, case–control, cohort and prospective clinical studies published between 1994 and 2024, with a combined patient population of 296,519, were included in this systematic review and meta-analysis [21,22,23,24,25,26,27,28,29,30,31,32,33,34,35,36,37,38,39,40,41,42,43]. The studies were conducted in 13 countries, predominantly Iran, Greece, Italy, Turkiye, Spain, Brazil, India and Mexico. Nine studies evaluated SNHL in RA, five in SLE, five in pSS and three in SSc. One study examined SNHL in both RA and pSS [33]. Sample size varied from small studies with 24–50 patients in hospital-based studies to large studies with more than 13,000 patients in nationwide studies. The mean age ranged from 30.9 to 64.6 years. Females accounted for 66.7–100% of the patient population, reflecting the epidemiology of the systemic SARDs. The number of patients with SNHL across studies ranged from 6 in cross-sectional studies to 145 in nationwide cohort studies (Table 1).

### 3.2. Quality Assessment

**Figure 2:**
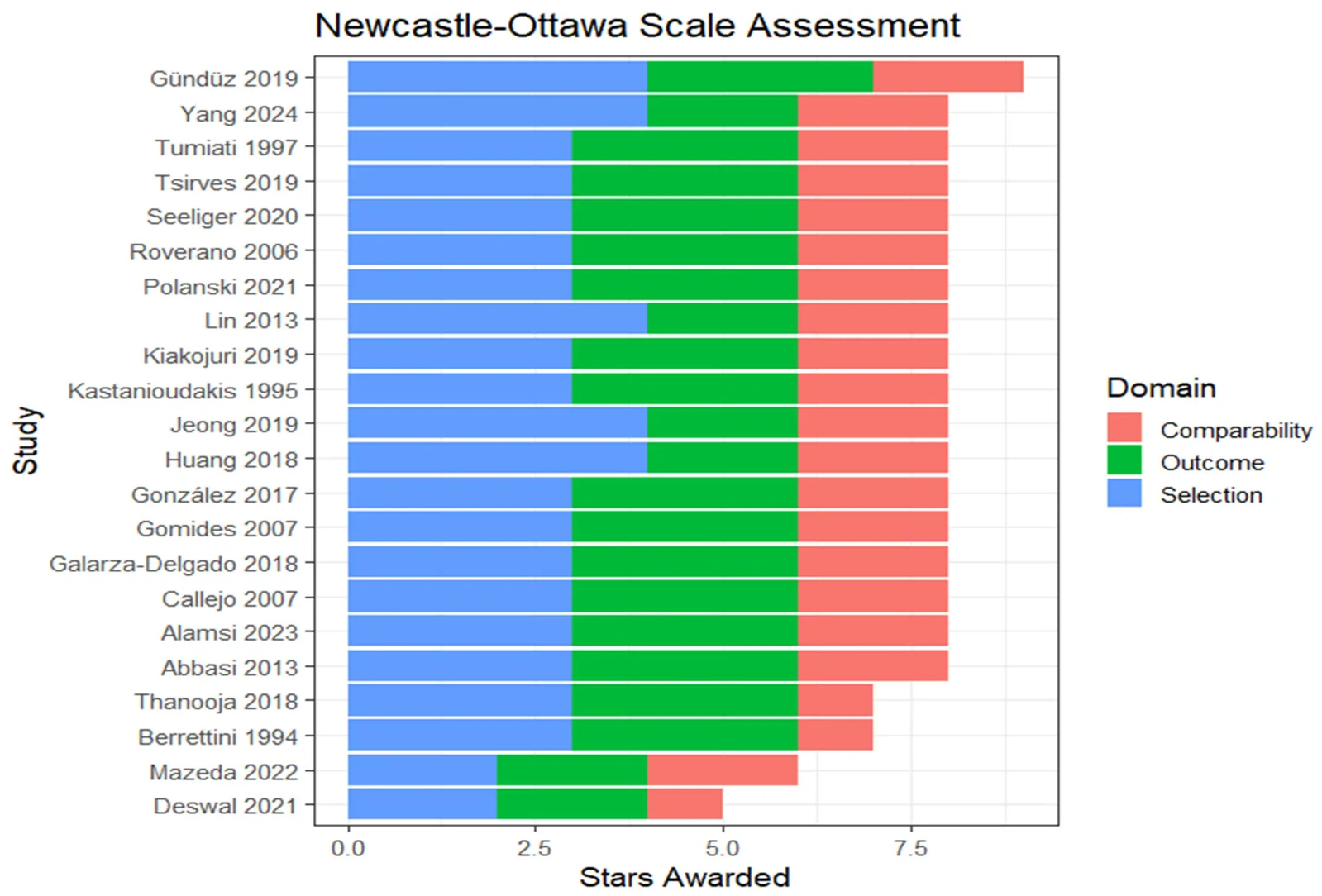
NOS risk of bias assessment of included studies [21,22,23,24,25,26,27,28,29,30,31,32,33,34,35,36,37,38,40,41,44,45].

The NOS results showed that overall risk of bias was low to moderate (Figure 2). Twenty studies were rated as good quality (7–9 stars) while two studies were rated as moderate quality (5–6 stars). Most studies scored highly for participant selection and outcome assessment, whereas several studies demonstrated insufficient adjustment for confounding factors, which represented the principal methodological limitation. No study was classified as poor quality.

### 3.3. Primary Outcomes

#### 3.3.1. Prevalence of SNHL

**Figure 3:**
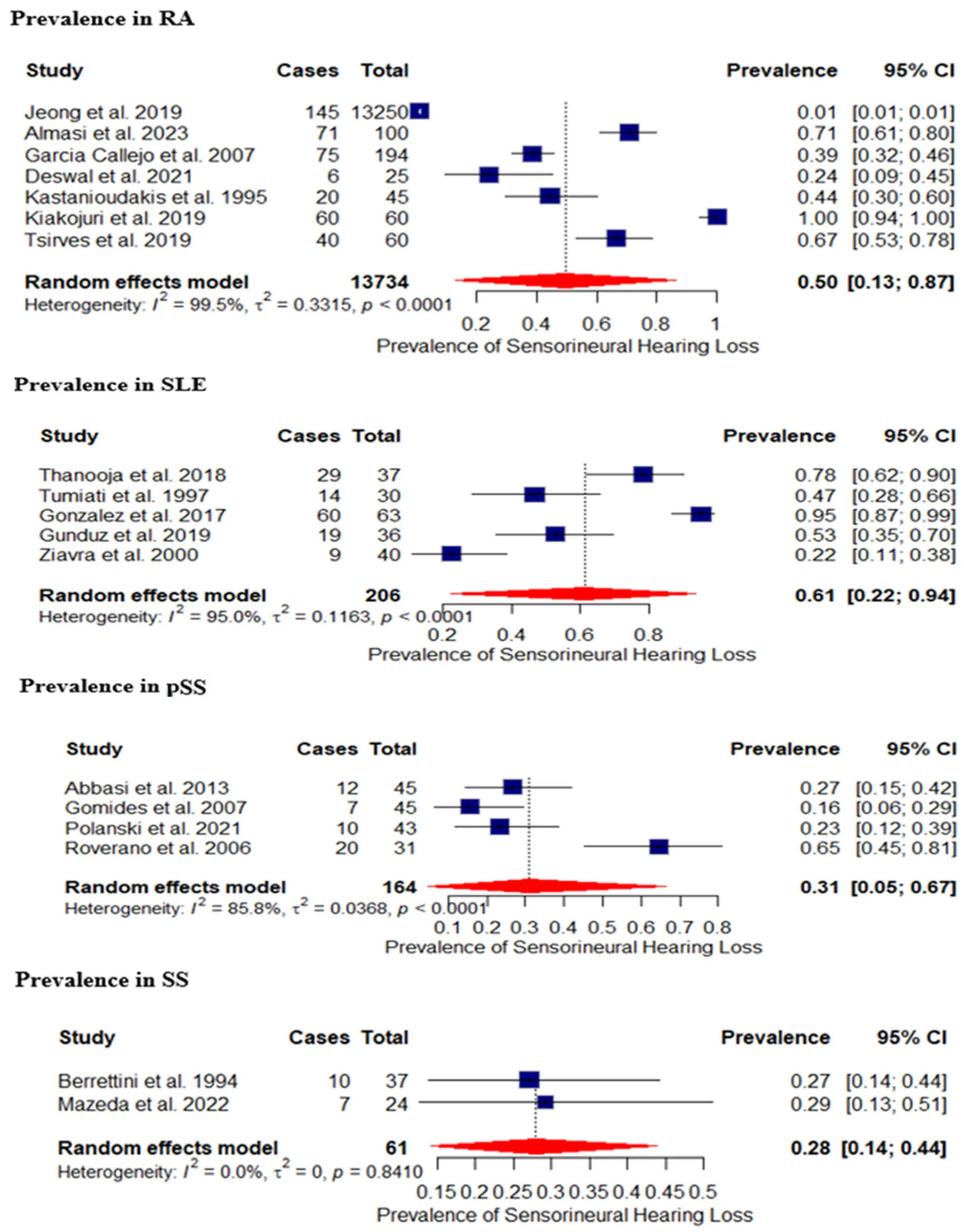
Forest plot showing prevalence of SNHL in patients with RA, SLE, pSS and SSc [21,22,23,24,25,29,30,31,33,34,35,36,37,38,39,40,41,44].

Eighteen studies [21,22,23,24,25,26,30,31,34,36,38,39,40,41,42] with a combined population of 14,165 were included in a meta-analysis of the prevalence of SNHL in patients with RA, SLE, pSS and SSc, and the results are shown in Figure 3. The pooled prevalence of SNHL was 50% (95% CI: 13–87%, *p* < 0.001) in patients with RA, with individual study estimates ranging from 1% in nationwide cohorts to 100% in a small clinical case–control study [26,34]. In SLE, the pooled prevalence was 61% (95% CI: 22–95%, *p* < 0.001) with individual study estimates ranging from 22% to 95% [40,41].

In pSS, the pooled prevalence was 31% (95% CI: 5–67%, *p* < 0.001), with individual study estimates ranging from 16% to 65% [23,25]. In SSc, the pooled prevalence was 28% (95% CI: 14–44%, *p* < 0.001), with individual study estimates ranging from 27% to 29% [35,37]. Substantial heterogeneity was reported in RA (*I*^2^ = 99.5%), SLE (*I*^2^ = 95.0%) and pSS (*I*^2^ = 85.5%) but was negligible in SSc (*I*^2^ = 0.0%). Overall, the high heterogeneity reflects differences in study design, patient characteristics, diagnostic criteria and audiological assessment methods.

#### 3.3.2. Odds Ratios of SNHL

**Figure 4:**
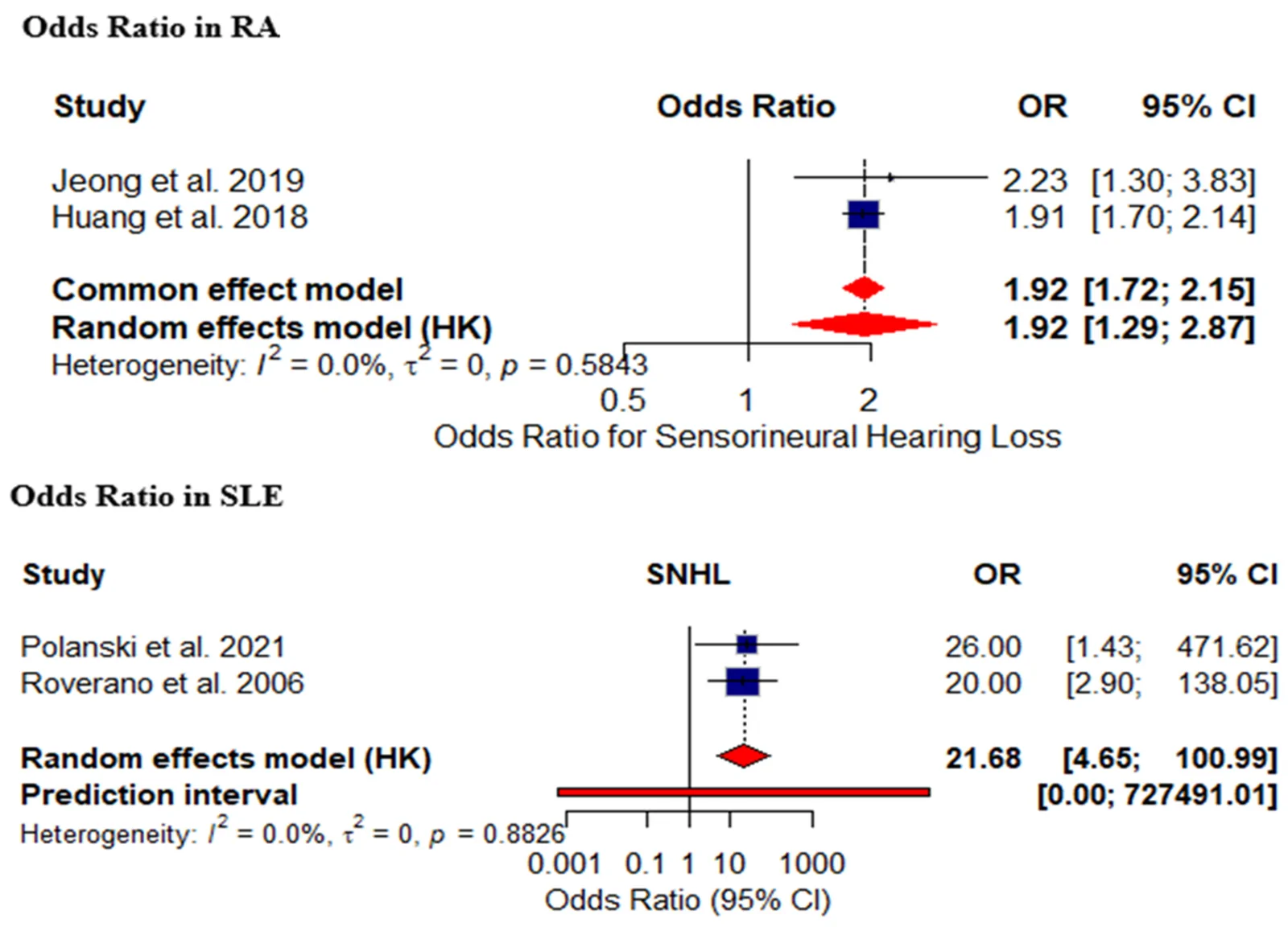
Forest plot showing odds ratios of SNHL in patients with RA and SLE [24,25,28,44].

Five studies [21,25,26,27,29] were included in the meta-analysis of the odds ratio of SNHL in RA and SLE compared to healthy controls. The results are shown in Figure 4. The pooled analysis yielded higher odds of SNHL in patients with in RA and SLE compared to healthy controls. In RA, the pooled odds ratio was 1.92 (95% CI: 1.29–2.87) while in SLE, the pooled odds ratio was 21.68 (95% CI: 4.65–100.99), with negligible heterogeneity (*I*^2^ = 0%). Pooled analyses of the odds ratio of SNHL in pSS and SS were not feasible. Only one study evaluated the association between pSS and SNHL, reporting a significantly higher odds ratios of OR = 1.72 (95% CI: 1.60–1.86) compared to healthy controls [27].

#### 3.3.3. Sub-Group Analysis

**Figure 5:**
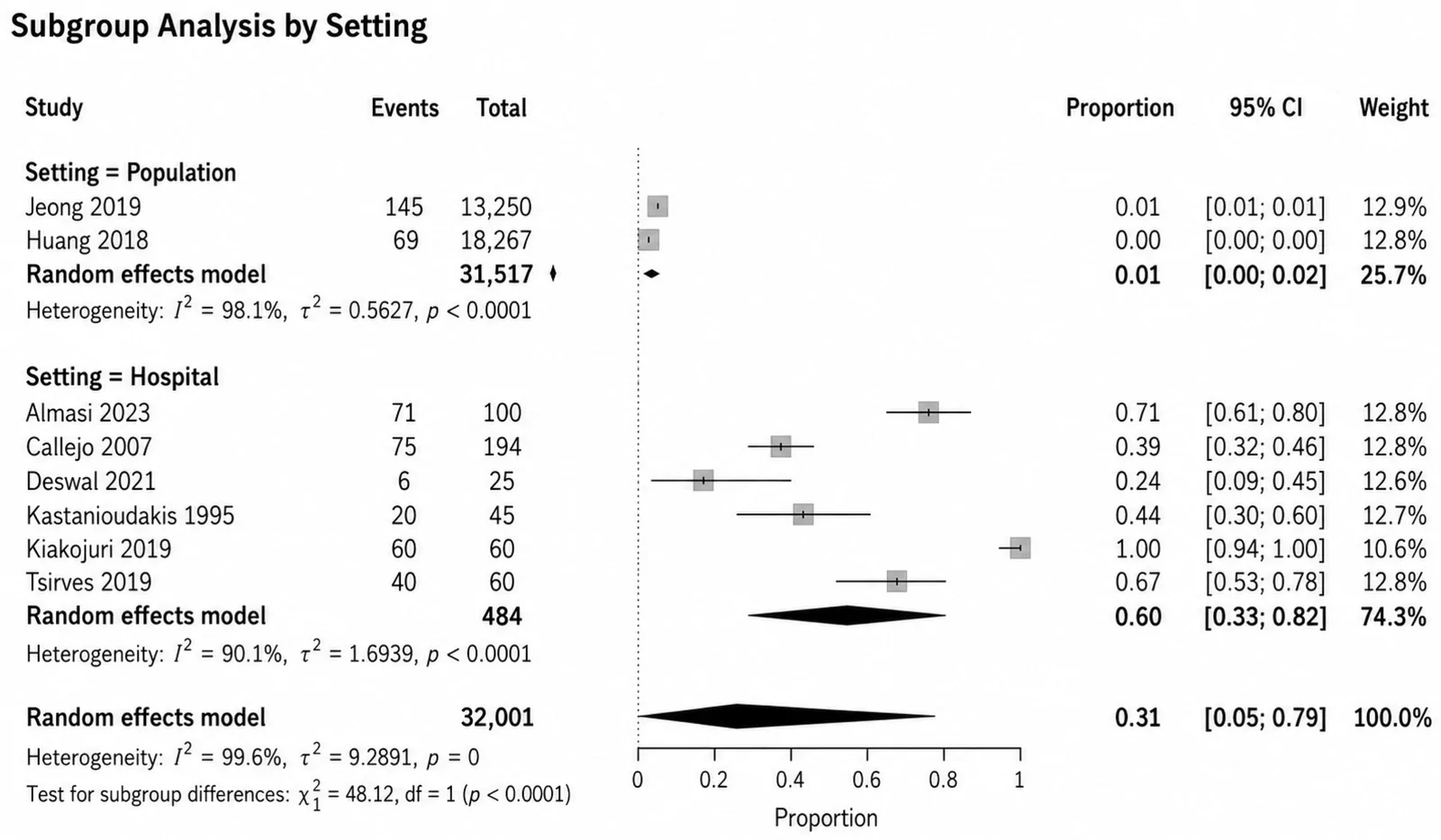
Forest plot showing subgroup analysis of pooled prevalence of SNHL in RA patients based on study setting (population vs. hospital-based) [23,25,28,29,30,31,33,35].

**Figure 6:**
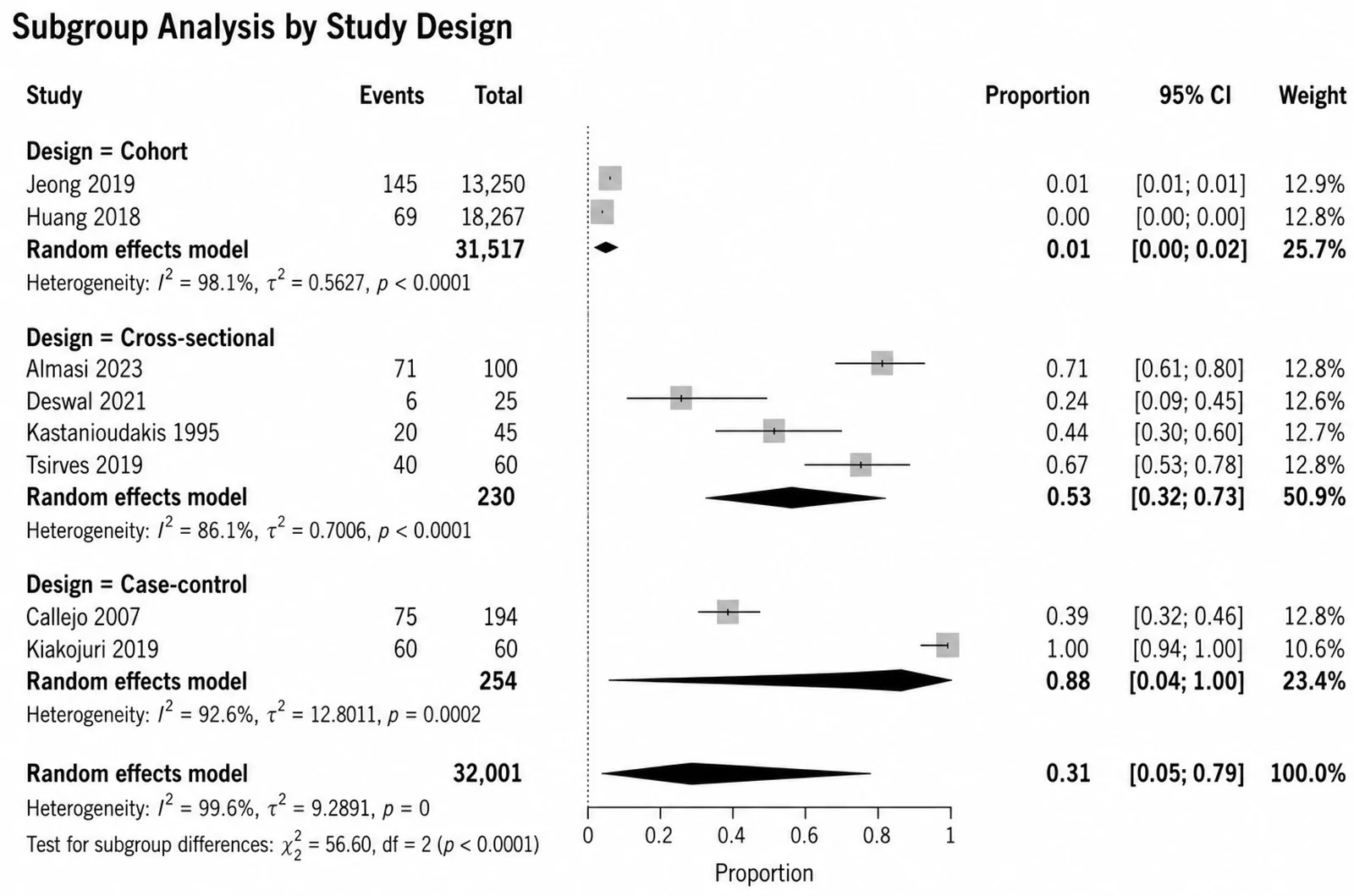
Forest plot showing subgroup analysis of pooled prevalence of SNHL in RA patients based on study design (cohort, vs. cross-sectional vs. case–control) [23,25,28,29,30,31,33,35].

**Figure 7:**
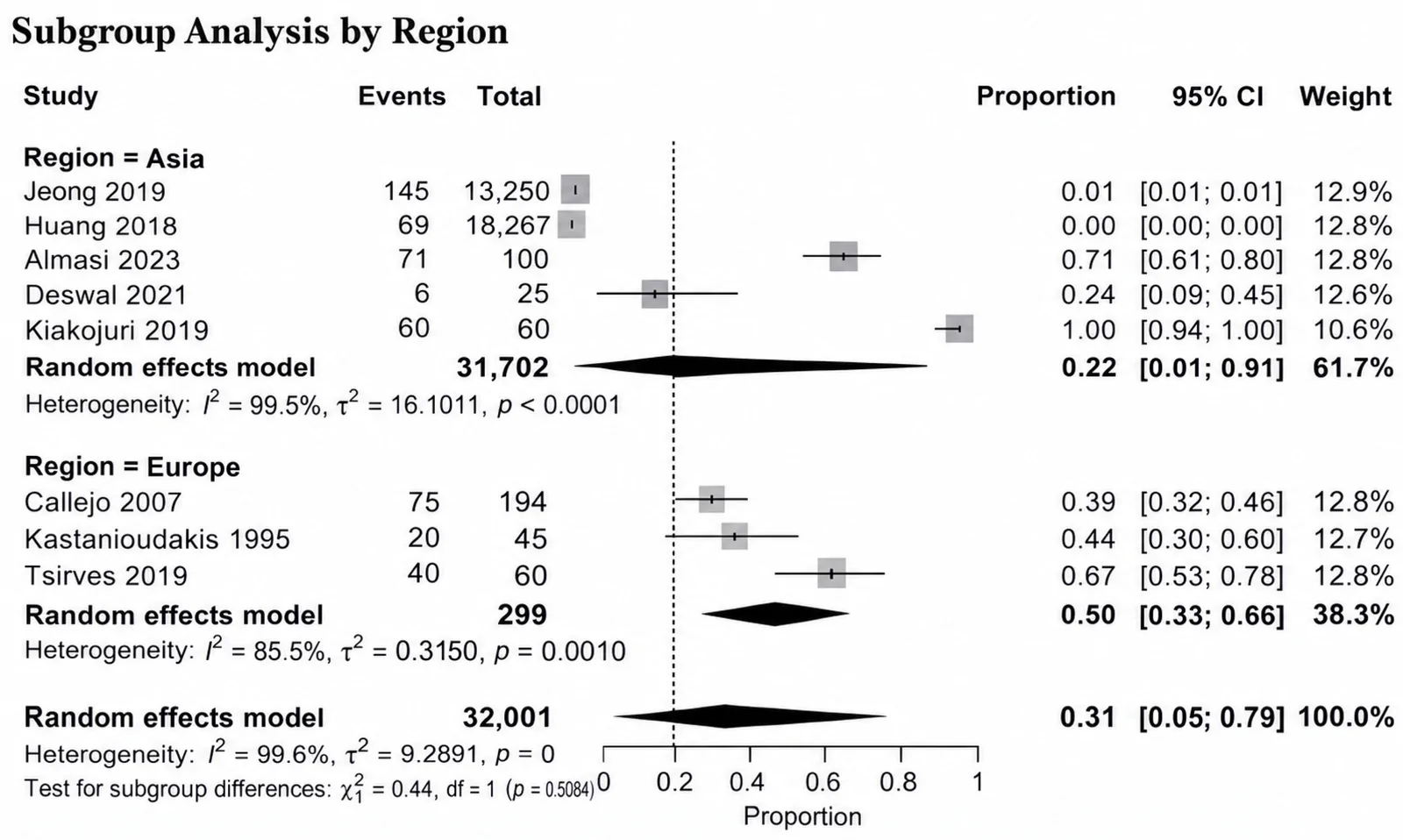
Forest plot showing subgroup analysis of pooled prevalence of SNHL in RA patients based on study region (Asia vs. Europe) [23,25,28,29,30,31,33,35].

Subgroup analysis of pooled prevalence of SNHL in RA based on study setting (Figure 5) demonstrated a significant difference between population-based cohorts versus hospital-based studies (X^2^ = 48.12, df = 1, *p* < 0.0001). Population-based cohort studies reported substantially lower pooled estimates of 1% (95% CI: 0–2%; *I*^2^ = 98.1%) whereas hospital-based studies reported markedly higher pooled estimates of 60% (95% CI: 33–82%; *I*^2^ = 90.1%). These findings suggest study setting was a major contributor of observed heterogeneity across studies. Subgroup analysis of pooled prevalence of SNHL in RA based on study design (Figure 6) showed significant differences among cohort, cross-sectional and case–control studies (X^2^ = 56.60, df = 2, *p* < 0.0001). Cohort studies yielded the lowest pooled prevalence (1%; 95% CI: 0–2%, *I*^2^ = 98.1), followed by cross-sectional studies (53%; 95% CI: 32–73%, *I*^2^ = 86.1%), and the highest was reported in case–control studies (88%; 95% CI:4–100%, *I*^2^ = 92.6%). The high pooled estimates in cross-sectional and case–control studies may reflect the variability in patient selection and ascertainment, suggesting that study design may significantly contribute to observed heterogeneity. A subgroup analysis of pooled prevalence estimates of SNHL in RA based on geographical region (Figure 7) did not demonstrate a statistically significant difference between studies conducted in Asia and Europe (X^2^ = 0.44, df = 1, *p* < 0.51). For Asian studies, the pooled prevalence was 22% (95% CI: 1–91%, *I*^2^ = 99.5%), while pooled prevalence was 50% (95% CI: 33–66%, *I*^2^ = 85.5%) for European studies. The lack of statistical significance and persistence of within-group heterogeneity suggested that the observed variability in prevalence estimates was not explained by geographical region.

#### 3.3.4. Meta-Regression

An exploratory univariable meta-regression analyses was performed to investigate whether study-level characteristics explained the variability in the reported prevalence of SNHL in RA (n = 8). The results are presented in Table 2. Sample size was the strongest contributor of between-study heterogeneity (β = −0.00034, SE = 0.00005, *p* < 0.001), explaining 89.2% of the variability observed. Both study setting (β = −5.371, SE = 0.925, *p* < 0.001) and study design (β = −6.329, SE = 1.523, *p* < 0.001) were also significantly associated with between-study heterogeneity, explaining 86.9% and 78.8% of the variability observed. Other study characteristics, like publication year, mean age and sex (female), were not significantly associated with variability in the prevalence of SNHL in RA (*p* > 0.05).

Meta-regression was not performed for SLE, pSS, or SSc because the limited number of studies available for each disease did not permit reliable moderator analyses. Further stratification would also have resulted in subgroups containing too few studies for meaningful quantitative comparison; therefore, potential sources of heterogeneity in these diseases were explored descriptively rather than through formal meta-regression.

#### 3.3.5. Sensitivity Analysis

Leave-out analysis demonstrated that pooled prevalence was robust across all diseases (Figure 5). Exclusion of any single study did not substantially change the pooled prevalence estimates or heterogeneity across studies for RA (*I*^2^ = 99.5–99.6%), SLE (*I*^2^ = 92.5–96.1%) or pSS (*I*^2^ = 85.8–90.5%).

#### 3.3.6. Publication Bias

**Figure 8:**
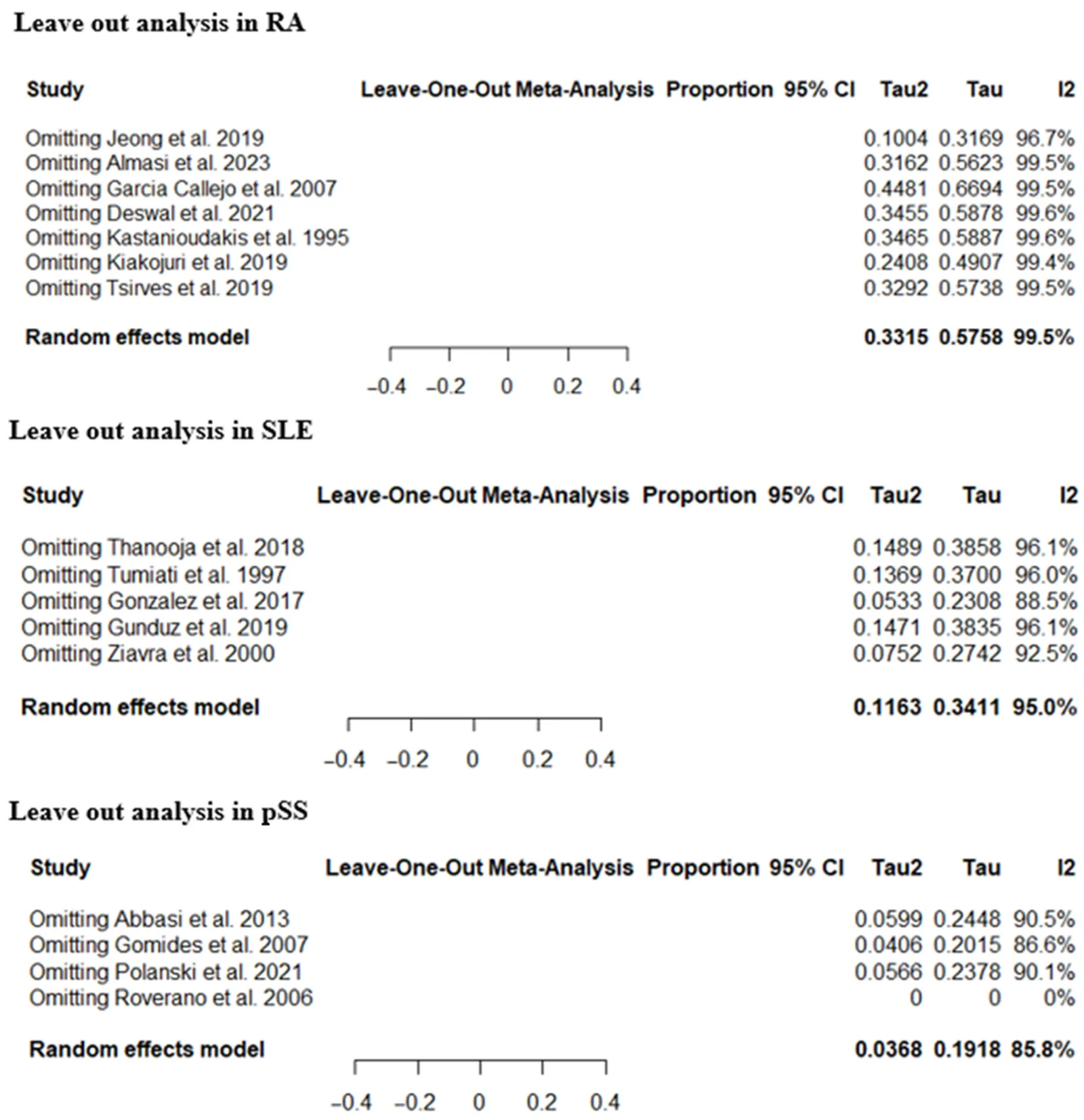
Leave-one-out-analysis of pooled prevalence of SNHL in patients with RA, SLE and pSS [21,22,23,24,25,29,30,31,33,35,37,38,39,40,41,44].

**Figure 9:**
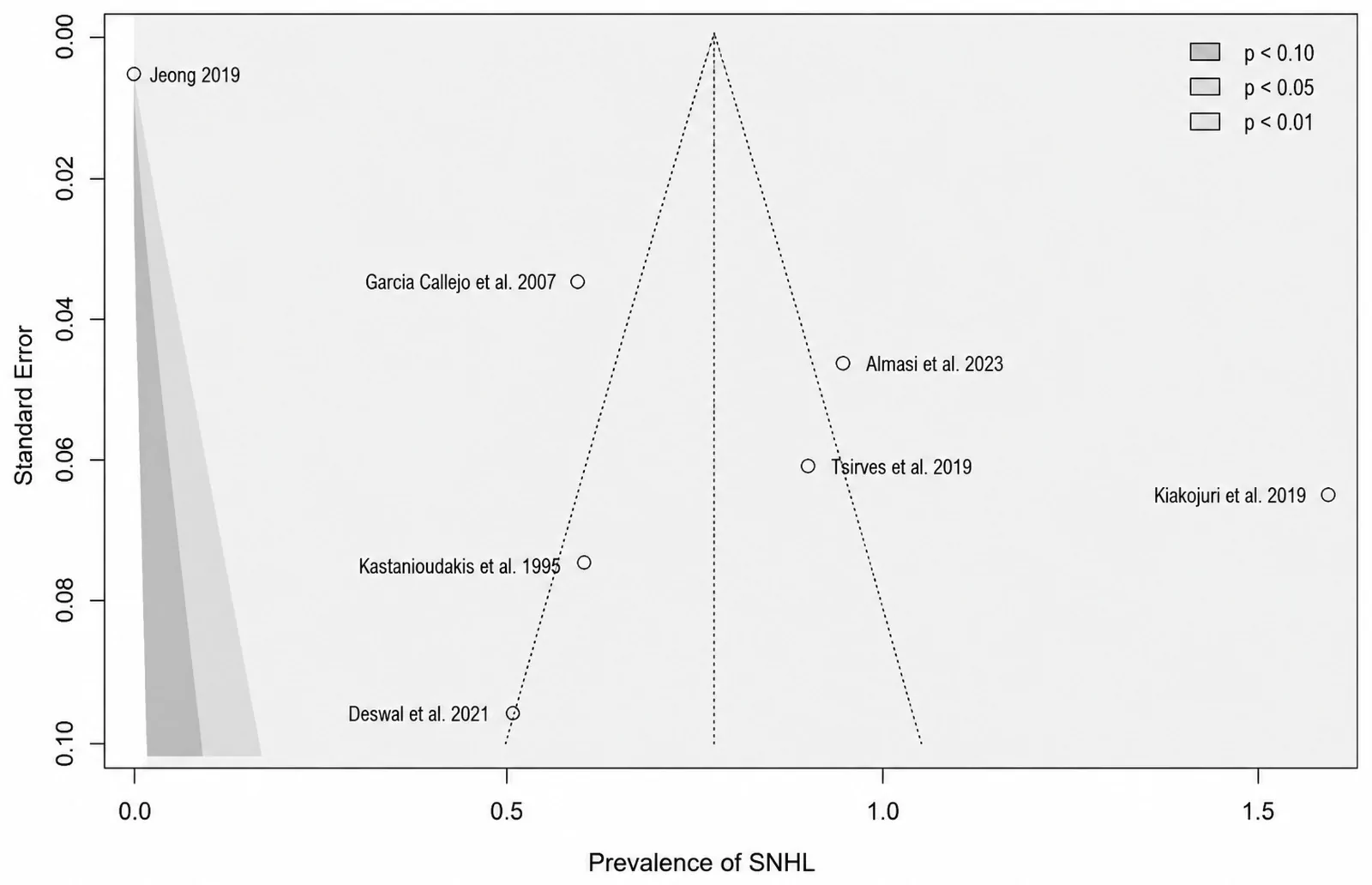
A funnel plot assessing potential publication bias among studies reporting the prevalence of SNHL in patients with RA [23,25,29,30,31,33,35].

A funnel plot to assess the publication bias among studies reporting the prevalence of SNHL in patients with RA was generated (Figure 8). The plot suggests possible asymmetry. However, the findings should be interpreted with caution, given the small number of studies (n = 7) and high heterogeneity, as asymmetry may reflect heterogeneity rather than publication bias (Figure 9).

#### 3.3.7. Certainty of Evidence

The certainty of the evidence for each of the primary outcome was assessed using GRADE framework, and the results are presented in Table 3. As all included studies were observational, the evidence was initially rated as low-certainty evidence. The certainty of evidence was not downgraded for risk of bias or indirectness as most studies demonstrated good methodological quality. However, the certainty of evidence was downgraded for inconsistency due to the substantial statistical heterogeneity observed and for imprecision where pooled estimates were based on a limited number of studies, small sample sizes or wide CIs. Publication of bias for pooled prevalence of SNHL in RA was considered undetected, as the funnel plot showed no evidence of small-study effects. For the remaining outcomes, publication bias could not be formally assessed for the outcomes because fewer than 10 studies were available.

### 3.4. Secondary Outcomes

#### 3.4.1. Audiometry Findings

Eighteen studies investigating SNHL in patients with systemic autoimmune rheumatic diseases reported audiometry findings (Table 4). Pure-tone audiometry consistently demonstrated elevated hearing thresholds among patients with systemic autoimmune rheumatic diseases. Abnormalities were predominantly reported in the high- and extended high-frequency ranges. Several studies reported significantly worsening speech discrimination, particularly in RA patients, while speech audiometry remained largely preserved in early to mild disease. Subclinical cochlear dysfunction was associated with extended high-frequency audiometry.

#### 3.4.2. Diagnostic Assessment

Across studies, tympanometry was largely normal (Type A), which suggested a primarily sensorineural rather than conductive source of hearing loss (Table 5). Several cohort studies reported acoustic reflex abnormalities, while otoacoustic emissions and audiometry brain response tests demonstrated dual pathology affecting both the cochlear and retrocochlear dysfunction, confirming sensorineural hearing loss [30,32,42].

#### 3.4.3. Prognostic Factors

Across studies, longer disease duration and greater disease activity were consistently associated with poorer hearing thresholds in RA [23,24,30], pSS [38,40,41] and SSs [37] patients (Table 6). There was no clear association between disease activity and hearing threshold in SLE patients [23,25]. In pSS patients, one study reported that disease activity, autoantibodies or treatment were not associated with hearing thresholds [40]. Increased risk of SNHL among RA patients was reported in several population-based cohorts with reported hearing loss of [26,29]. Antimalarial therapy was associated with better high-frequency hearing in SLE patients, as reported in one study [21]. In contrast, hearing loss was not significantly associated with disease activity or treatment exposure [21].

## 4. Discussion

This systematic review and meta-analysis demonstrated that sensorineural hearing loss is a common extra-articular manifestation of systemic autoimmune rheumatic diseases, although prevalence varies considerably across the diseases and patient populations. The findings showed that the pooled prevalence estimates of SNHL in patients with RA, SLE, pSS and SSc were 50%, 61%, 31% and 28%, respectively. Patients with RA and SLE demonstrated increased odds of developing SNHL compared to healthy controls. Across studies, SNHL predominantly affected the inner ear, manifested in bilateral or extended high-frequency range and was frequently mild or subclinical during the early stages of the disease. Longer disease duration and greater disease activity were consistently associated with poorer hearing thresholds in patients with RA, pSS and SSc. Antimalarial therapy was associated with better high-frequency hearing in SLE patients. These findings confirm a moderate to high prevalence and increased risk of SNHL in patients with systemic autoimmune rheumatic diseases. Substantial heterogeneity was observed in RA, SLE and pSS, ranging from 85 to 99%, but was negligible in SS (*I*^2^ = 0%). Meta-regression further demonstrated that sample size, study setting and study design explained the heterogeneity.

The relatively high prevalence of SNHL observed in RA is plausible and likely reflects multiple pathogenic mechanisms underlying the disorders. Chronic systemic inflammation in RA is driven by persistent circulating immune complexes and elevated pro-inflammatory cytokines, including tumor necrosis factor-alpha (TNF-α), interleukin-6 (IL-6), interleukin-β. These mediators can bypass the blood–labyrinth barrier and directly target the stria vascularis and cochlear hair cells, driving localized oxidative stress, systemic tissue injury and cochlear dysfunction [5,7,11,12,13].

Microvascular injury represents another important mechanism. The labyrinthine artery, which supplies blood to the cochlear nerve, has minimal collateral circulation. A vasculitic or thrombotic insult of the artery, even when minor, may compromise cochlear perfusion, resulting in irreversible hair cell damage [12,23,34]. Immune-complex deposition and autoimmune-mediated vasculitis can target microvessels of the inner ear, restricting blood flow and contributing to progressive hearing loss due to oxygen deficits and tissue damage within the cochlear [12,25]. Experimental studies confirm that prolonged oxidative stress, triggered by noise exposure, increases damage to outer hair cells, leading to permanent SNHL [7,27,37]. These mechanisms were supported by the findings in this review. Elevated pure-tone thresholds and normal tympanometry were reported in most RA studies, supporting autoimmune cochlear rather than a conductive mechanism [33,35,41]. In several studies, extended high-frequency audiometry tests detected subclinical cochlear damage before it impaired conventional frequencies [21,32,33]. These findings confirm that structural degradation progresses systematically with chronic inflammatory activity and underscore the need for early diagnosis to prevent permanent hearing loss.

The audiometric findings observed across the autoimmune rheumatic diseases further supports early cochlear involvement. Pure-tone audiometry was strongly associated with elevated hearing thresholds, particularly at frequencies above 4 kHz, while extended high-frequency audiometry identified abnormalities at 9–16 kHz [33,41]. Speech discrimination remained relatively preserved in early stages of the diseases [23,32,34]. Tympanograms were frequently normal across studies, further suggesting that SNHL arises from cochlear pathology rather than middle-ear disease. Together, these findings are consistent with early-stage cochlear stress and degeneration of outer hair cells. Routine assessment of patients with SARDs should incorporate high-frequency audiometry particularly those with longstanding disease or chronic inflammatory activity.

Although evidence on prognostic factors remains scarce, the available evidence suggests an association between prolonged inflammatory burden and progressive cochlear dysfunction. Longer disease duration contributes to worse hearing thresholds in patients with RA and pSS [24,30,40] while greater inflammatory activity was directly linked with severe hearing loss [40,41]. These observations confirm that persistent systemic inflammation contributes to progressive inner ear injury over time. Interestingly, evidence in SLE was inconsistent. Better high-frequency hearing was observed in patients receiving antimalarial therapy [21] but there was no clear association between disease activity and hearing threshold [25].

The high heterogeneity across studies was further investigated for RA, for which the number of available studies permitted an exploratory quantitative analyses. Subgroup analyses demonstrated substantially lower prevalence estimates in population-based than hospital-based studies and lower estimates in cohort than cross-sectional or case–control studies. Exploratory meta-regression similarly identified sample size, study setting, and study design as significant study-level moderators of prevalence in RA. These findings should not, however, be quantitatively extrapolated to SLE or pSS. The limited numbers of studies available for these diseases precluded sufficiently reliable disease-specific meta-regression or further subgroup analyses, as stratification would have generated subgroups containing very few studies. Accordingly, the substantial heterogeneity observed in SLE and pSS remains incompletely explained, and differences in patient selection, disease characteristics, SNHL definitions, diagnostic thresholds, and audiometric protocols should be considered plausible rather than quantitatively confirmed sources of heterogeneity.

The apparently large association between SLE and SNHL should be interpreted with particular caution. The pooled OR of 21.68 was derived from only two studies and had a very wide 95% confidence interval (4.65–100.99), indicating considerable uncertainty regarding the true magnitude of the association. Accordingly, although the available studies suggest an increased occurrence of SNHL among patients with SLE compared with controls, the evidence is insufficient to establish a precise magnitude of risk. Consistent with this uncertainty, the certainty of evidence for this outcome was rated as very low using GRADE.

### 4.1. Clinical Implications

The findings of this review have several clinical implications for clinical professionals. ENT specialists should consider SNHL as an important extra-articular manifestation of autoimmune rheumatic diseases rather than an incidental comorbidity. Our findings show that hearing loss in patients with SARDs is frequently mild, bilateral, and limited to the high-frequency range, hence the reliance on hearing-related symptoms alone may delay diagnosis underestimate the real burden of the diseases. Rheumatology patients who report hearing difficulties, vestibular symptoms or unexplained bilateral high-frequency should be evaluated for underlying hearing impairment. Baseline and periodic audiologic assessment is recommended for patients with SARDs, especially with longstanding disease or persistent inflammatory activity to detect cochlear dysfunction before patients develop clinically significant hearing loss. Audiologic assessment remain important evaluation tools for SNHL and can aid in early detection of inner ear cell damage. Early detection of cochlear involvement may facilitate timely hearing rehabilitation and optimize immunomodulatory therapy, reducing long-term morbidity and improving quality of life. ENT physicians should consider high-frequency audiometry even in asymptomatic patients with longstanding autoimmune disease.

### 4.2. Limitations

This review has several limitations that should be acknowledged. Heterogeneity was very high for a pooled prevalence analysis, reflecting substantial variability in study design, patient selection, diagnostic methods, and audiometric protocols. Most studies included in the review were cross-sectional and observational studies, which may have limited causal reference. Reporting of effect estimates was inconsistent across studies, limiting the pooled analysis of prevalence and odds ratios of SNHL for the various diseases. The small sample sizes in some studies may have limited the generalizability of the findings. Further, the few studies available for pooled and odds ratios in several SARDs limited the assessment of publication bias for specific diseases.

An additional source of heterogeneity was the lack of uniformity in the definition and assessment of SNHL across the included studies. Studies differed in audiometric methods, frequencies evaluated, and diagnostic thresholds used to define hearing loss, which may have resulted in differences in case ascertainment and contributed to variability in prevalence estimates. Clinical heterogeneity among the included populations should also be considered, as patients differed in disease duration, disease activity, treatment exposure, and potentially relevant vascular or thrombotic manifestations, including vasculitis and antiphospholipid syndrome. Because these disease characteristics were not consistently reported across studies, their independent contribution to hearing loss could not be reliably evaluated. Therefore, the pooled prevalence estimates should be interpreted cautiously as summary estimates across clinically and methodologically heterogeneous populations.

Furthermore, the limited number of studies available for SLE, pSS, and SSc precluded reliable disease-specific meta-regression and more granular subgroup analyses, leaving the sources of heterogeneity in these diseases incompletely explained.

## 5. Conclusions

This systematic review and meta-analysis demonstrates that SNHL is reported across rheumatoid arthritis, systemic lupus erythematosus, primary Sjögren syndrome, and systemic sclerosis, although prevalence estimates vary substantially among studies and should be interpreted in the context of considerable clinical and methodological heterogeneity. Audiometric findings indicate that hearing abnormalities are frequently bilateral and may predominantly involve high or extended high frequencies. Comparative evidence supports increased odds of SNHL in RA, whereas the large pooled association observed in SLE is based on limited, very-low-certainty evidence and remains imprecise. Evidence regarding comparative risk in pSS and SSc is currently insufficient for robust conclusions. Future prospective multicenter studies using standardized definitions of SNHL and uniform audiometric protocols are needed to establish more reliable disease-specific prevalence and risk estimates and to clarify prognostic factors associated with hearing impairment in systemic autoimmune rheumatic diseases.

## Figures and Tables

**Table 1:** Characteristics of the included studies.

Author	Country	Study Design	SARD Type	Sample Size (n)	Mean Age (Years)	Sex (F) %	SNHL (n)
Jeong et al. 2019 [25]	Korea	Nationwide cohort	RA	13,250	≥20	66.65	145
Huang et al. 2018 [28]	Taiwan	Retrospective cohort	RA	18,267	53.6	78.4	NR
Almasi et al. 2023 [31]	Iran	Cross-sectional	RA	100	53.95	78	55–61
Callejo et al. 2007 [30]	Spain	Case–control	RA	194	40.3	72.5	75
Deswal et al. 2021 [35]	India	Cross-sectional	RA	25	NR	100	6
Galarza-Delgado et al. 2018 [32]	Mexico	Cross-sectional	RA and pSS	30	47.5	100	NR
Kastanioudakis et al. 1995 [29]	Greece	Prospective clinical study	RA	90	52.5	93.3	40
Kiakojuri et al. 2019 [33]	Iran	Case–control	RA	60	47.2	91.7	60
Tsirves et al. 2019 [23]	Greece	Cross-sectional	RA	60	64.6	81.7	40
Abbasi et al. 2013 [21]	Iran	Case–control	SLE	45	34.9	91.1	12
Gomides et al. 2007 [22]	Brazil	Cross-sectional	SLE	45	30.9	100	7
Lin et al. 2013 [27]	Taiwan	Population cohort	SLE	7168	35.7	88.42	NR
Polanski et al. 2021 [44]	Brazil	Cross-sectional	SLE	43	40.8	97.6	10
Roverano et al. 2006 [24]	Argentina	Cross-sectional	SLE	31	35	100	20
Thanooja et al. 2018 [37]	India	Prospective observational	pSS	37	45.8	97.3	29
Tumiati et al. 1997 [38]	Italy	Cross-sectional	pSS	30	55	100	14
González et al. 2017 [40]	Mexico	Cross-sectional	pSS	63	49.5	95.2	60
Gündüz et al. 2019 [41]	Türkiye	Case–control	pSS	36	51.4	100	19
Yang et al. 2024 [26]	Taiwan	Population cohort	pSS	20,266	52.1	73.1	NR
Berrettini et al. 1994 [36]	Italy	Cross-sectional	SSc	37	48.7	81.1	10
Mazeda et al. 2022 [34]	Portugal	Cross-sectional	SSc	24	58.4	79.2	7
Amor-Dorado et al. 2008 [42]	Spain	Case–control	SSc	31	63.3	94	27

Abbreviation: RA—Rheumatoid Arthritis; SLE—Systemic Lupus Erythematosus; pSS—Primary Sjogren Syndrome; SSc—Systemic Sclerosis; NR—Not Reported.

**Table 2:** Meta-regression of potential sources of heterogeneity in prevalence of SNHL in RA.

Moderator	β	SE	*p*-Value	R^2^ (%)
Sample size	−0.00034	0.00005	<0.001	89.2
Study setting (population-based vs. hospital-based)	−5.371	0.925	<0.001	86.9
Study design (cohort, vs. cross-sectional vs. case–control)	−6.329	1.523	<0.001	78.8
Publication year	−0.011	0.134	0.932	0.0
Mean age	−0.041	0.199	0.839	0.0
Female (%)	0.124	0.100	0.216	5.8

β: Regression coefficient; SE: standard error; R^2^: proportion of between-study heterogeneity explained by the moderator.

**Table 3:** Summary of the findings and GRADE assessment of the certainty of evidence for the primary outcomes.

Outcome	No. of Studies	Sample (n)	Pooled Effect (95% CI)	Heterogeneity (I^2^)	Certainty of Evidence (GRADE)	Key Findings
SNHL prevalence in RA	8	32,001	50% (13–87%)	99.5%	Very low	Approximately half of patients with RA presented SNHL, although estimates varied substantially across studies.
SNHL prevalence in SLE	5	7332	61% (22–95%)	95.0%	Very low	SNHL was common among patients with SLE, but heterogeneity was considerable.
SNHL prevalence in pSS	6	20,432	31% (5–67%)	85.8%	Very Low	Approximately one-third of patients with pSS had SNHL, with high variability across studies.
SNHL prevalence in SSc	3	92	28% (14–44%)	0%	Very low	SNHL affected approximately one-quarter of patients with SS, with findings consistent across studies.
Odds of SNHL in RA	2	97,835	OR 1.92 (1.29–2.87)	0%	Low	Patients with RA had nearly twice the odds of developing SNHL compared with healthy controls.
Odds of SNHL in SLE	2	74	OR 21.68 (4.65–100.99)	0%	Very low	Patients with SLE demonstrated markedly increased odds of SNHL, although the estimate was imprecise given small number of studies and wide confidence interval.
Odds of SNHL in pSS	1	81,064	OR 1.72 (1.60–1.86)	Not applicable	Low	Patients with pSS had significantly increased odds of SNHL compared with controls.

**Table 4:** Audiometry findings reported in studies evaluating SNHL in patients with SARDs.

Study	Disease	Pure-Tone Audiometry	Speech Audiometry
Almasi et al., 2023 [31]	RA	Air-conduction thresholds vs. controls (*p* < 0.001); largest difference at 8000 Hz (32.19 vs. 10.0 dB right; 35.5 vs. 11.0 dB left).	Speech discrimination was significantly lower in RA (93.6% vs. 96.1%; *p* = 0.03).
García Callejo et al., 2007 [30]	RA	Elevated air-conduction thresholds across conventional frequencies with significantly worse four-frequency PTA than controls.	
Deswal et al., 2021 [35]	RA	Air-conduction thresholds elevated at 250–8000 Hz; bone-conduction thresholds elevated at 500–8000 Hz.	
Galarza-Delgado et al., 2018 [32]	RA/pSS	Extended high-frequency audiometry (9–16 kHz) showed higher thresholds in RA (40.1 dB) and pSS (43.7 dB) than controls (28.5 dB).	
Kastanioudakis et al., 1995 [29]	RA	PTA demonstrated significantly higher air- and bone-conduction thresholds compared to controls.	Speech audiometry demonstrated reduced speech discrimination in RA patients.
Kiakojuri et al., 2019 [33]	RA	Higher thresholds at 2, 4 and 8 kHz (14.1, 16.9 and 23.0 dB) compared with controls.	Speech discrimination was significantly reduced.
Tsirves et al., 2019 [23]	RA	Average hearing level significantly higher than controls (25.1 vs. 17.8 dB right; 26.3 vs. 18.0 dB left).	
Abbasi et al., 2013 [21]	SLE	Air-conduction thresholds are higher at 250, 500, 2000, 4000 and 8000 Hz; bone-conduction thresholds are elevated at 250, 500 and 4000 Hz.	
Gomides et al., 2007 [22]	SLE	Pure-tone audiometry identified predominantly mild SNHL affecting 15.6% of patients.	
Polanski et al., 2021 [44]	SLE	PTA was similar between antimalarial users and non-users except at 8000 Hz, where untreated patients had worse thresholds.	No clinically important difference in word-recognition performance
Roverano et al., 2006 [24]	SLE	High-frequency SNHL (15–60 dB), predominantly affecting 2–8 kHz.	
Thanooja et al., 2018 [37]	pSS	Mean air-conduction threshold at 8000 Hz: 29.5 dB (right), 28.4 dB (left); bone-conduction threshold at 4000 Hz: 21.9 dB (right), 20.5 dB (left).	
Tumiati et al., 1997 [38]	pSS	SNHL was identified in 46%, predominantly with high-frequency sloping audiograms.	
González et al., 2017 [40]	pSS	Extended high-frequency thresholds elevated at 9–16 kHz (51.7 dB right; 50.9 dB left).	
Gündüz et al., 2019 [41]	pSS	Thresholds are significantly higher across conventional and very-high frequencies; the mean very-high-frequency threshold is approximately 45 dB.	Speech discrimination scores were slightly reduced compared with controls.
Ziavra et al., 2000 [39]	pSS	Pure-tone audiometry identified cochlear SNHL mainly involving high frequencies.	Speech audiometry is normal in most patients; used to characterize hearing impairment.
Berrettini et al., 1994 [36]	SSc	Pure-tone audiometry identified SNHL in 10 patients and mixed hearing loss in four.	Speech audiometry confirmed cochlear dysfunction.
Mazeda et al., 2022 [34]	SSc	Hearing loss in 37.5%; SNHL in 29.2%.	Speech audiometry demonstrated worse speech discrimination in patients with hearing loss.

**Table 5:** Diagnostic assessment performed in studies evaluating SNHL in patients with SARDs.

Study	SARD Type	ABR/OAE/Tympanometry	Vestibular Assessment
Almasi et al. [31]	RA	Type A tympanogram and elevated acoustic reflexes are identified in a minority of RA patients.	
Kastanioudakis et al. [29]	RA	Type A tympanogram in most ears; occasional Type As and Type C patterns.	Acoustic reflex testing
Kiakojuri et al. [33]	RA	Predominantly Type A tympanograms; few Type As.	
Tsirves et al. [23]	RA	Type A tympanogram in most ears; absent stapedius reflexes in some patients.	Acoustic reflexes
Gomides et al. [22]	SLE	Tympanometric abnormalities were identified in a small proportion of patients.	
Thanooja et al. [37]	pSS	Type A tympanogram in 83.8%; absent acoustic reflexes in 18.9%.	
González et al. [40]	pSS	Normal Type A tympanograms in >95% of ears.	
Gündüz et al. [41]	pSS	TEOAE performed; reduced static compliance and elevated acoustic reflex thresholds.	Medial olivocochlear efferent system
Ziavra et al. [39]	pSS	ABR was performed when clinically indicated; tympanometry was performed in all patients.	
Berrettini et al. [36]	SSc	Tympanometry performed.	Vestibular testing
Mazeda et al. [34]	SSc	Abnormal tympanometry and absent acoustic reflexes were reported in a subset of patients.	

**Table 6:** Prognostic factors associated with SNHL in patients with SARDs.

Study	SARD Type	Prognostic Factor Evaluated	Main Finding
Jeong et al. [25]	RA	RA diagnosis (population cohort)	RA is associated with increased risk of sudden SNHL (HR 2.229).
Huang et al. [28]	RA	RA diagnosis	RA is independently associated with hearing loss (HR 1.91).
Kastanioudakis et al. [29]	RA	Disease duration; treatment	Longer disease duration associated with worse hearing thresholds.
Tsirves et al. [23]	RA	Disease activity	Higher DAS28 is associated with poorer hearing thresholds.
Gomides et al. [22]	SLE	Disease duration; disease activity	No significant association was demonstrated.
Polanski et al. [44]	SLE	Antimalarial therapy	Antimalarial users had better high-frequency hearing.
Roverano et al. [24]	SLE	Disease activity; treatment	No clear association identified.
Thanooja et al. [37]	pSS	Disease duration	Longer disease duration associated with increased hearing impairment.
González et al. [40]	pSS	Disease activity	Higher disease activity is associated with worse extended high-frequency thresholds.
Ziavra et al. [39]	pSS	Disease duration, disease activity, autoantibodies, treatment	SNHL is associated only with disease duration; no association with disease activity, autoantibodies or treatment.
Berrettini et al. [36]	SSc	Disease duration	Longer disease duration associated with cochlear involvement.

## Data Availability

The data that support the findings of this study are available from the corresponding author, Dr. Alaa Safia, upon reasonable request.

## Supplementary Materials

The following supporting information can be downloaded at: https://www.mdpi.com/article/10.3390/jcm15166456/s1, Table S1: PRISMA checklist.
